# Neuronavigated Repetitive Transcranial Ultrasound Stimulation Induces Long-Lasting and Reversible Effects on Oculomotor Performance in Non-human Primates

**DOI:** 10.3389/fphys.2020.01042

**Published:** 2020-08-19

**Authors:** Pierre Pouget, Stephen Frey, Harry Ahnine, David Attali, Julien Claron, Charlotte Constans, Jean-Francois Aubry, Fabrice Arcizet

**Affiliations:** ^1^Institute of Brain and Spinal Cord, UMRS 975 Inserm, CNRS 7225, UMPC, Paris, France; ^2^Rogue Research Inc., Montreal, QC, Canada; ^3^Physics for Medicine Paris, Inserm, ESPCI Paris, CNRS, PSL Research University, Univ Paris Diderot, Sorbonne Paris Cite, Paris, France; ^4^Université de Paris, Institute of Psychiatry and Neuroscience of Paris (IPNP), Inserm U1266, Team Pathophysiology of Psychiatric Disorders, Paris, France; ^5^GHU Paris Psychiatrie et Neurosciences, Site Sainte-Anne, Service Hospitalo-Universitaire, Paris, France; ^6^Institut de la Vision CNRS, Inserm, Sorbonne Université, Paris, France

**Keywords:** focused ultrasound, neurostimulation, neuronavigation, brain, transcranial, non-human primates

## Abstract

Since the late 2010s, Transcranial Ultrasound Stimulation (TUS) has been used experimentally to carryout safe, non-invasive stimulation of the brain with better spatial resolution than Transcranial Magnetic Stimulation (TMS). This innovative stimulation method has emerged as a novel and valuable device for studying brain function in humans and animals. In particular, single pulses of TUS directed to oculomotor regions have been shown to modulate visuomotor behavior of non-human primates during 100 ms ultrasound pulses. In the present study, a sustained effect was induced by applying 20-s trains of neuronavigated repetitive Transcranial Ultrasound Stimulation (rTUS) to oculomotor regions of the frontal cortex in three non-human primates performing an antisaccade task. With the help of MRI imaging and a frame-less stereotactic neuronavigation system (SNS), we were able to demonstrate that neuronavigated TUS (outside of the MRI scanner) is an efficient tool to carry out neuromodulation procedures in non-human primates. We found that, following neuronavigated rTUS, saccades were significantly modified, resulting in shorter latencies compared to no-rTUS trials. This behavioral modulation was maintained for up to 20 min. Oculomotor behavior returned to baseline after 18–31 min and could not be significantly distinguished from the no-rTUS condition. This study is the first to show that neuronavigated rTUS can have a persistent effect on monkey behavior with a quantified return-time to baseline. The specificity of the effects could not be explained by auditory confounds.

## Introduction

Transcranial Ultrasound Stimulation (TUS) is a safe, focused and non-invasive method of brain stimulation that has emerged as a novel and valuable tool for studying brain function in humans and animals. The use of TUS gathered momentum in the first decade of the new millennium, using low frequency, low intensity ultrasound waves to stimulate rodent primary motor cortex that generated motor responses (Yoo et al., 2011; King et al., 2013; Younan et al., 2013; Li et al., 2016; Ye et al., 2016) without damaging brain tissue (Yoo et al., 2011). In the same vein, TUS has been shown to immediately alter electromyographic and electroencephalographic measurements in sheep (Lee et al., 2016) and humans (Legon et al., 2014; Lee et al., 2016), and suppress somatosensory evoked potentials in swine (Dallapiazza et al., 2018). Recently, our group has shown in awake non-human primates that single pulses of TUS can modulate visuomotor behavior (Deffieux et al., 2013) with brief modulating effects (∼100 ms) from TUS in single neuronal responses (Wattiez et al., 2017). Whether such modulations due to TUS can be temporally extended and controlled remains to be demonstrated.

Accurate transducer positioning is crucial when sonicating target sites for desired stimulation effects. Presently, TUS studies of higher cognitive function demonstrate high inter-subject and between-group variability since anatomical variability and function may not correlate well in many brain areas (Brett et al., 2002). Clearly, localization of a subject’s individual anatomical brain region as well as precise placement and angle of the transducer is indispensable for successful targeting. However, skull differences as well as individual variability of the cerebral sulci have shown variations of up to 20 mm in the different axes, with some electrode positions having larger variability than others (Herwig et al., 2003; Okamoto et al., 2004). A different method is sometimes used to position the TMS coil by employing a functional-guided approach. If the TMS effect on the performance of a certain behavioral task is known, this task can serve as a “functional” probe to position the coil with subsequent targets or tasks (Göbel et al., 2001). Such “hunting” procedures can, however, be time-consuming due to the fact that different locations need to be tested by trial-and-error. Furthermore, the interpretation of the results with respect to brain anatomy is limited by the fact that the coil position is determined functionally and not anatomically.

**FIGURE 1:**
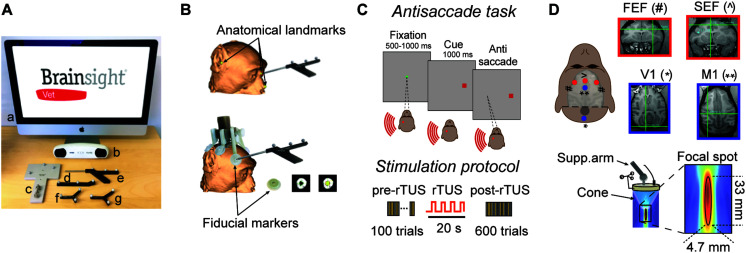
Experimental set-up. **(A)** List of navigation tools to carry out a monkey registration: Brainsight computer (a), position sensor (b), calibration block (c), tool tracker (d), navigation pointer (e), subject tracker (f), and transducer tracker (g). **(B)** Subject registration. Top: registration can be based on facial landmarks which are defined as the bridge of the nose, the tip of nose and the notch above the tragus of the ears. Bottom: In the present study, we used fiducial markers which are fixed to the headpost of the subject resulting in higher co-registration accuracy between the subject and the MRI scan. These fiducial markers create hyperintensities on the MR images that are easily distinguishable. **(C)** Antisaccade paradigm. The animal starts the trial by fixating a central spot (green square) for 500–1000 ms. A peripheral cue (a red square at 16 degrees of eccentricity) is then presented either at the left or right of the visual field on the horizontal meridian for 1000 ms. The animal is rewarded by making an eye movement to the opposite location of the peripheral cue (antisaccade). The brain area stimulated by rTUS is indicated by the red circle (here, left FEF). Each animal practices a block of 100 trials before rTUS (pre-rTUS block) and then six post-rTUS trials. Each block contains 100 trials (50 right and 50 left) and lasts on average 5 min. The rTUS train lasts 20 s at a frequency of 10 Hz with stimulation pulses of 320 kHz. **(D)** Experimental set-up. The ultrasonic transducer is manually guided toward the Region of Interest (ROI) by a neuro-navigation system (Brainsight Vet). The cigar-shaped focal stimulation (bottom panel) targeted the FEF and SEF location [indicated by red dots: FEF (#) ans SEF (∧)]. We also performed sham stimulation sessions by placing the transducer over the primary motor or visual cortex [V1 (*) and M1 (**) indicated by blue dots]. The insets represent the MRI images used to locate ROI (red squares) and control areas (blue squares). The intersection of horizontal and vertical green lines in each quadrant indicates the point at which the focused ultrasound stimulations were directed.

To better account for inter-individual anatomical differences, image-guided frameless stereotaxic neuronavigation systems (SNS) have been used with Transcranial Magnetic Stimulation (TMS) in both humans and non-human primates (Schoenbaum et al., 2003; Sparing et al., 2007; Gerits et al., 2011). SNS use the subject’s individual MRI for navigation via a subject-image co-registration procedure based on facial/cranial landmarks. Although there can be technical limitations to this procedure as the quality of the MRI and limitations of the position sensor, the targeting error is within several millimeters (Neggers et al., 2004; Schönfeldt-Lecuona et al., 2005). In non-human primates, the accuracy of transducer placement can be improved to within 1 mm when using rigid fiducial markers during the co-registration of the subject to their scan (Frey et al., 2004; Figure 1A). SNS have been used in several ways: first, the gross anatomy of the cerebral cortex itself may serve as a reference system. However, reliable anatomical landmarks exist for very few target sites and individual differences of gyral folding and cortical layering have to be taken into consideration. The functional identification of a brain area with SNS requires that the same individual participates in both the fMRI and TUS study. Positioning of the coil over the local maxima of the fMRI activation cluster no longer assumes a fixed relationship between anatomical landmarks and task-related functional activations. Alternatively, functional neuroimaging data obtained from the literature can be used for navigation (“probabilistic approach”) (Paus et al., 1997). Although there are undoubtably slight anatomical variabilities amongst inter-individual regions in primate brain there is still predictable functional anatomical regions within primates (Amiez et al., 2019); this method takes advantage of and that has a relatively high consistency in the location of task-related “activations” across individuals.

Many studies optimized TMS setups in order to induce a modulatory effect which outlasts the stimulation, either for stand-alone treatments (Rossini et al., 2015), or combined with neuroimaging (Bestmann et al., 2008; Siebner et al., 2009).

Producing inhibitory effects that can outlast the period of stimulation for minutes or hours is crucial for rehabilitative purposes (Rossi and Rossini, 2004). It is typically achieved with repetitive stimulation (Wassermann and Lisanby, 2001; Hoffman and Cavus, 2002), each repeated stimulation increasing the magnetic energy delivered to the target. Increasing the acoustic energy is constrained by thermal considerations, as acoustic energy can be transferred to tissue (McDannold et al., 2010) and bone (Pinton et al., 2011), especially when considering that numerical models showed that the minimum activation energy was six orders of magnitude higher to elicit an action potential with ultrasound as compared to direct current (Krasovitski et al., 2011; Plaksin et al., 2014). Using repetitive pulses of ultrasound decreases the thermal rise (Constans et al., 2018). For these reasons, repetitive pulses have been proposed here and tested.

In this study, we present a detailed account of the effects of repetitive Transcranial Ultrasound Stimulation (rTUS) on the oculomotor regions of awake macaque monkeys while the animals were performing an antisaccade task. In primates, the frontal eye fields (FEF) and the supplementary eye fields (SEF) are two sophisticated cortical brain areas that play important roles in the control of visual attention and eye movements (Schall, 2015). Electrophysiological approaches exploring these areas have provided causal evidence about the role of the FEF, with direct bearing on oculomotor functions (Bruce and Goldberg, 1985), while a more indirect role has been proposed for the SEF (Schall, 2004, 2015). In addition to seeking new visual targets, an important part of saccadic behavior is to suppress eye movements that would be made to novel but behaviorally irrelevant stimuli. To investigate such control of voluntary vs. reflexive saccades, a test paradigm called the antisaccade task has been developed (Condy et al., 2005). In this task the subject is required to suppress a saccade toward a stimulus that appears in their periphery but instead must generate a voluntary saccade toward the opposite visual field (antisaccade).

In humans and macaques, functional imaging studies have shown that both the FEF and SEF are activated bilaterally during antisaccades (Schlag-Rey et al., 1997; Brown et al., 2006, 2007). Furthermore, patients with FEF lesions display a normal percentage of errors on the antisaccade task, but their correct antisaccades exhibit an increased latency (Pierrot-Deseilligny et al., 1991). Stimulation over FEF with single pulses of TUS or TMS have reported saccade latencies to be significantly delayed (Grosbras and Paus, 2002; Deffieux et al., 2013). These results have been interpreted as a modulation of saccadic inhibition in the contralateral visual field due to disruption of neural processing in the stimulated FEF area. Using repetitive TMS (rTMS), previous studies have described facilitating effects (Crevits et al., 2005) when compared to single pulse parameters (Olk et al., 2005), speculating that repetitive stimulation may more strongly interfere with the processing of inhibition, particularly active in antisaccades compared to single pulses (Everling and Munoz, 2000). Our current results establish rTUS as an innovative neurostimulation methodology by showing (1) that rTUS induced a reduction of saccade latencies only on ipsilateral antisaccades; (2) that this effect was reversible with a return to baseline after 18–31 min; and (3) that the latency decrements were dependent on neurostimulation locations.

## Materials and Methods

### The Stereotaxic Navigation System (SNS)

The Brainsight Vet stereotaxic navigation system (SNS, Figure 1A) was created by Rogue Research Inc. (Qc, Canada), and is composed of a standard computer with the neuronavigation software, an optical position sensor (Vicra-Northern Digital Inc., Ont, Canada), an articulated arm to hold the transducer, a subject and transducer tracker, a navigation pointer, as well as a calibration block for registering the acoustic focus of the transducer. Once the subject’s MRI data are transferred to the navigation computer, 3D reconstructions of the brain are created, atlas spaces can be introduced (e.g., MNI or Tailairach), and regions of interest are selected along with trajectories to brain targets. The primary reason for using the Brainsight navigation system was to aid in sonicating intended brain regions, and although TUS is seen as a non-invasive procedure, reducing the number of unnecessary stimulation sessions. An important advantage in using the MRI guided navigation system is that targets and trajectories to targets can be planned well in advance of the actual experimental procedures; a refinement to existing sonication protocols. During a navigated TUS session, the researcher is able to refer to the saved sonication locations in real-time on the MRI reconstructed images of the subject, simplifying data collection.

### Subject Registration

There are generally two methods to register the subject to their high resolution T1-weighted MRI brain scan prior to a neuronavigated TUS procedure. The first is based on skin landmarks while the second method uses a fiducial marker system that is rigidly attached to the animal’s skull. The first procedure, mostly used with human subjects, identifies anatomical landmarks on the 3D reconstructed skin within the software and homologous points on the subject’s skin prior to a sonication session. Recommended landmarks are defined as the bridge of the nose, the tip of nose, and the notch above the tragus of the ears (Figure 1B). Unfortunately, the accuracy of this type of registration leads to an error of approximately 2–5 mm with one of the difficulties being identifying the exact landmark on both the skin and the MRI scan (Ruohonen and Karhu, 2010). This method can also be used for non-human primate subjects. A second procedure is to fix fiducial markers on the subject before the MR scan; this method is typically more accurate as the landmarks are rigid. The present study used rigid markers for registration and is the most common method used with neuronavigation with non-human primate researchers. Prior to MRI, the animal is anesthetized and fitted with an MRI compatible head-fixation implant to ensure head immobilization during sonication. Attached to this headpost is an adaptor that is fitted with MRI compatible disks that hold adhesive radio-opaque fiducial markers (Figure 1B). These fiducial markers create hyperintensities on the MR images (Figure 1B) and are also easily identified on the animal. The neuronavigation software uses the positions of these fiducial markers to compute the coordinates of the neuronavigated TUS session and are necessary for subsequent co-registrations of the subject to the MR scan. The fiducial markers are only needed for the MRI and during TUS registration in the testing suite. This method has an error of approximately 1 mm based on phantom MRI tests (Frey et al., 2004). In order to calibrate the focal point of the transducer, a tool tracker is rigidly fixed to the TUS device. A jig is placed on the Brainsight calibration block to hold the transducer steady above the calibration pin in front of the optical position sensor (Figure 1A). Once calibrated, the TUS transducer can be moved over the head of the subject corresponding to desired pre-planned stimulation sites inside of the brain. The corresponding locations are visualized and updated in real-time on the computer monitor as the transducer is navigated into the correct orientation and trajectory of the desired stimulation sites.

### Animals

Data were collected from three captive-born male macaques (one *Macaca fascicularis*; monkey G, 8 kg; two *Macaca mulatta*; monkey L and monkey S, 13, and 8.5 kg, respectively). Monkeys were paired-housed and handled in strict accordance with the recommendations of the Weatherall Report on good animal practice. Monkey housing conditions, surgical procedures and experimental protocols were all carried out in strict accordance with the National Institutes of Health guidelines (1996) and the recommendations of the EEC (86/609) and the French National Committee (87/848). Our experiments were authorized by the Animal Health and Veterinary Medication Division of the Department of Public Veterinary Health, Nutrition and Food Safety of the French Ministry of Health (last renewal N°: DTPP 2010-424). Our routine laboratory procedures included an environmental enrichment program where monkeys had access to toys, mirrors and swings. Monkeys also had visual, auditory and olfactory contact with other animals and, when appropriate, could touch and groom each other.

Under isoflurane and aseptic conditions, we surgically implanted a titanium headpost on each animal. Any possible pain associated with surgeries was pharmacologically ameliorated by means of a daily injection of ketofen (0.03 ml/kg) or buprenorphine (0.067 ml/kg). An institutional veterinarian regularly monitored the environment and wellbeing of the monkeys. Before participating in this study, animals had been periodically chaired, head-posted and trained to perform a series of tasks for a period of 3–6 months, until they became regular and proficient performers.

### Experimental Apparatus for Non-human Primate Antisaccade Task Sessions

The animals were seated in a primate chair (Crist Instrument, Hagerstown, MD, United States) with their head fixed in a darkened booth. Animals were positioned 52 cm in front of a 60 Hz monitor. Eye movements were recorded with an infrared eye tracker (Eyelink 1k, SR-Research, Ont, Canada), and eye position was digitized and sampled at 1000 Hz and stored for off-line analysis. Visual paradigms and data acquisition were controlled by a computer running a real-time data acquisition system (Rexeno software; for further details see Krasovitski et al., 2011). Saccades were detected using custom Matlab scripts that first searched for significantly elevated velocity (>30°s^–1^). Saccade initiation and termination were then defined as the beginning and end of the monotonic change in eye position lasting 12 ms before and after the high-velocity gaze shift.

Prior to the first experimental session, animals were specifically trained in an anti-saccadic paradigm (Figure 1C), in which they were required to initially fix on a central brown target. Between 500 and 1000 ms after fixation onset, simultaneous to the disappearance of the central brown stimulus, and without a time gap, a red square appeared for 1000 ms at a location 16° to the right or left of it. Monkeys were trained not to look at this peripheral target but instead initiate a saccade in the opposite direction as soon as possible (i.e., an antisaccade). Monkeys were rewarded if the saccade fell within a 12°× 12° window centered at the mirror location of the visual target. Failure to trigger a saccade within 1000 ms after target onset canceled the trial and was considered an error trial. To analyze the performance in the antisaccade task, we divided the behavioral data into unambiguous categories. An eye movement centered in the mirror location of the 12°× 12° window visual target was defined as a correct trial, any other eye movement was defined as an error. The antisaccade task was chosen since prior human and monkey TMS experiments have revealed prosaccade paradigms to be much less sensitive to single pulse TMS interference than antisaccades (Olk et al., 2005). We computed saccade latency on rewarded antisaccades trials only and all non-rewarded trials were excluded from these latency comparisons.

### Positioning the rTUS Transducer

We used a single element focused ultrasound transducer (H115, Sonic Concept, Bothell, WA, United States; central frequency 250 kHz, diameter 64 mm, FD# 1) in the current experiment. Even though the manufacturer specifies a 250 kHz central frequency, the transducer can be operated at 250, 320, 850, and 1380 kHz (Constans et al., 2017). We decided to use a 320 kHz frequency, as previously validated for modulating non-human primates behavior (Deffieux et al., 2013). A coupling cone (C103, Sonic Concepts, Bothell, WA, United States) filled with degassed water was placed between the transducer and the animal’s head (Figure 1D). The transducer was fixed on a mechanical arm with four rotational axes (Viewmaster LCD, Osmond Ergonomics, Wimborne, United Kingdom) to enable flexible positioning of the transducer over the head. A thin layer of echographic gel (Aquasonic 100, Parker Laboratories Inc., Fairfield, NJ, United States) was applied to the skin and the membrane of the coupling cone to ensure acoustic coupling (Figure 1D). Based on simulations done in water at 320 kHz, the −6 dB ovoidal focal spot was cigar shaped with a size of 4.7 × 4.7 × 33 mm (Figure 1D).

### Acoustic and Thermal Modeling

The acoustic wave propagation of our focused ultrasound protocol was simulated using a k-space pseudospectral method-based solver, k-Wave (Cox et al., 2007) to obtain estimates for the pressure amplitude, peak intensity, spatial distribution, and thermal impact at steady state. 3D maps of the skull were extracted from a monkey CT scan (monkey L (Constans et al., 2017), 0.14 mm slice resolution, 0.33 mm slice distance). Water acoustic values were ρ_water_ = 1000 kg.m^−3^ and c_water_ = 1500 m.s^−1^ and soft tissues were assumed to be homogeneous, with the following acoustic values: *ρ*_tissue_ = 1030 kg.m^−3^ and c_tissue_ = 1560 m.s^−1^.

In the bone, a linear relationship between the Hounsfield Units (HU) from the CT scan and the sound speed, as well as the density, was used. The power law model for attenuation was $\alpha_{\text{att}}=\alpha_{\min}+\alpha_{\max}^{*}\Phi^{\beta }$ where the porosity Φ is defined by $\Phi =\frac{\rho_{\max}-\rho }{\rho_{\max}-\rho_{\text{tissue}}}$ in the skull (Aubry et al., 2003). The attenuation coefficients for the acoustic propagation α_min_ and α_max_ depended on the frequency: αmin = α_min⁡0_*f*^b^ with α_min0_ = 0.2dB.cm^−1^.MHz^−b^ and α_max_ = α_max⁡0_*f*^b^ with α_max⁡0_ = 8dB.cm^−1^.MHz^−b^ (Aubry et al., 2003). We set the parameters to ρ_max_ = 2200kg.m^−3^, c_max_ = 3100m.s^−1^ (Younan et al., 2013), β = 0.5 (Aubry et al., 2003), b = 1.1 (Constans et al., 2018). The attenuation coefficients in bone accounted for both absorption and scattering (Pinton et al., 2011). In a consistent manner, in soft tissues, attenuation coefficient for the acoustic propagation α_tissue_ depended on the frequency: α_tissue_ = α_tissue0_*f*^b^ with α_tissue0_ = 0.6dB.cm^−1^.MHz^−b^.

The propagation simulation was performed at 320 kHz with a 150 μs-long pulse signal (enough to reach a steady state). The transducer was modeled as a spherical section (63.2 mm radius of curvature and 64 mm active diameter). The acoustic focus of the transducer was positioned on the target (Left FEF). The simulated pulses were spatially apodised (*r* = 0.35) on the spherical section. Ultrasound propagates first through water before entering the skull cavity with the geometrical focal point located below the surface, inside the brain. Simulations were performed in free water, and the maximum amplitude obtained was used to rescale the results in skull. The thermal modeling is based on the bio-heat equation (Legon et al., 2014):

$$\rho \text{C}\frac{\partial \text{T}}{\partial \text{t}}=\kappa \nabla^{2}\text{T}+\text{q}+\text{w}\rho_{b}{\text{C}}_{\text{b}}\left(\text{T}-{\text{T}}_{\text{a}}\right)$$

where T, *ρ*, C, κ, and q are the temperature, density, specific heat, thermal conductivity and rate of heat production, respectively. Heat production is defined as $\text{q}=\alpha_{\text{abs}}\frac{P^{2}}{2\rho C}$, α_abs_ being the absorption coefficient and P the peak negative pressure. κ is set to 0.528 W.m^–1^.K^–1^ in soft tissue and 0.4 W.m^–1^.K^–1^ in the skull; C is set to 3600 J.kg^–1^.K^–1^ in soft tissue and 1300 J.kg^–1^.K^–1^ in the skull. In the tissue, the absorption coefficient was set to $\alpha_{\mathrm{abs max}}=\frac{\alpha_{0}}{3}=2.7\mathrm{dB}.\mathrm{cm}.{\text{MHz}}^{-\text{b}}$ (Goss et al., 1979). In the skull the longitudinal absorption coefficient is proportional to the density with $\alpha_{\mathrm{abs max}}=\frac{\alpha_{0}}{3}=2.7\mathrm{dB}.\mathrm{cm}.{\text{MHz}}^{-\text{b}}$ (Pinton et al., 2011). The last term corresponds to the perfusion process: w,ρ_b_,C_b_, and T_a_ correspond to the blood perfusion rate, blood density, blood specific heat and blood ambient temperature, respectively. These parameters are assumed homogeneous over the brain, although a more detailed description of the brain cooling processes can be found in the literature (Wang et al., 2016). The perfusion parameters are based on previous reports (Pulkkinen et al., 2011): w = 0.008 s^–1^; *r*_b_ = 1030 kg.m^–3^; C_b_ = 3620 J.kg^–1^.K^–1^ and T_a_ = 37°C.

The bioheat equation is solved by using a 3D finite-difference scheme in MATLAB (Mathworks, Natick, United States) with Dirichlet boundary conditions. Initial temperature conditions were 37°C in the brain, skull and tissue, and 24°C in the water coupling cone. Simulations were run over 1 min pre-sonication, followed by 20 s of sonication, and 5 min post-sonication, closely following the experimental procedure.

To quantify the pressure amplitude, peak intensities, spatial distribution, and potential temperature changes in the monkey brain associated with the TUS protocol targeting the FEF used in this study we simulated the acoustic wave propagation and its thermal effect in a whole head finite element model based on a high-resolution monkey CT scan. As estimated by these numerical simulations, the maximum spatial-peak pulse-averaged intensity (I_sppa_) at the acoustic focus point was 21.2 W/cm^2^ for the left FEF target (spatial peak temporal average intensities, I_spta_: 6.4 W/cm^2^). Given that the skull is more acoustically absorbing than soft tissue, the highest thermal increase is located in the skull itself, estimated by the simulation to be 1.53°C. For an approximate 0.5 mm thickness of the dura the maximum temperature in the brain below the dura was 37.4°C. The maximal thermal increase at the geometrical focus of the sonic transducer was less than 0.1°C.

### Repetitive Transcranial Ultrasound Stimulation Parameters

The ultrasound frequency was set to 320 kHz. The pulse duration was 30 ms with a rise and fall time set to 1 ms to avoid abrupt changes in pressure that generate brief white noise. The pulse repetition frequency was set to 10 Hz and the total sonication time was 20 s. The signal was generated by a TiePie generator (Handyscope HS5). A 75-watt amplifier (75A250A, Amplifier Research, Souderton, PA) was used to deliver the required power to the transducer and the input voltage of the transducer was monitored using a voltage probe (P6139A, Tektronix, Melrose, MA) connected to a TiePie oscilloscope. The amplifier gain was set to deliver an output voltage V_out_ = 173 V peak-to-peak, corresponding to a pressure amplitude of 0.76 MPa in water (calibrated with an in-house heterodyne interferometer; Constans et al., 2017). In order to estimate the skull attenuation of the ultrasound beam, a clean and degassed primate (*Macaca mulatta*) skull specimen was placed in front of the transducer in a degassed water tank and the pressure at the focus was estimated using a custom-built heterodyne interferometer (Royer and Casula, 1994) which uses a laser beam to detect the vibration of the ultrasound wave on a Mylar membrane. The amplitude of the vibration is then converted to pressure with a 10^–4^ Å/(Hz)^1/2^ sensitivity and a 20 MHz bandwidth (Royer and Dieulesaint, 1986). The transmission of the pressure through the degassed primate skull was assessed at seven different points arbitrarily chosen on the skull. The transmission ratio was found to be 58 ± 8% (standard deviation). The *in-situ* pressure delivered to the monkey brain transcranially was subsequently estimated at 0.44 ± 0.06 MPa. The equivalent mechanical index (MI) value was 1.3 with an intensity spatial peak pulse average (ISPPA) of 19 W/cm^2^ in free water. These values were attenuated to MI = 0.8 ± 0.1 and ISPPA = 6.5 ± 1.8 W/cm^2^, respectively inside the primate skull. Taking into account the pulse duration and PRF (respectively, 30 ms and 10 Hz, corresponding to a 30% duty cycle) during the neuro-stimulation sequence, the intensity spatial peak time average (ISPTA) was estimated to be 5.7 W/cm^2^ in free water and 1.9 W/cm^2^ inside the primate skull.

We targeted the FEF, SEF, cortical motor, and visual cortex with the navigated transducer using Brainsight Vet neuronavigation (Rogue Research Inc., QC, Canada). For each animal, the target areas were identified based on the anatomical MRI data. The FEF was identified as the fundus of the arcuate sulcus in front of the spur, along the anterior bank of the arcuate sulcus (Schall et al., 2009) and SEF neurostimulation site was anatomically centered along the dorso-medial part of the frontal convexity cortex (Parthasarathy et al., 1992). In monkey L, the target ROI was placed in the right FEF, whereas the control was placed in the right hemisphere of the visual cortex (V1). In monkey S, the target ROI was set in the left FEF and the control region in the left hemisphere of the visual cortex (V1). In monkey G, the target ROI was set in the SEF of the left hemisphere, while the control region was set closer to the ROI in motor cortex (left M1) due to the large mass of muscle on the animal’s skull. SEF, motor and visual cortex areas were defined on the MRI visually and according to stereotaxic coordinates (Figure 1D).

### Experimental Protocol

In this study, animals performed a total of 40 sessions (10 stimulated sessions “rTUS” and 10 non-stimulated sessions “no-rTUS” in the regions of interest “ROIs” and in the control regions). Each experimental session contained one practice block, followed by a total of 7 blocks of 100 antisaccades (50 on each side randomly distributed). Navigated repetitive Transcranial Focused Ultrasound Stimulation (rTUS) was only delivered after the first block of trials (1 of 7) was completed. In this study, the first block is referred to as pre-rTUS and the six following blocks as post-rTUS (Figure 1C). In order to keep conditions across all experimental sessions as similar as possible, the transducer was placed on the target regions (ROI and control) in both rTUS and non-rTUS sessions.

### Data Analysis

Saccade latencies were compared across different factors: (1) the side of the antisaccade (i.e., contralateral or ipsilateral to the stimulation location), (2) rTUS stimulation or no stimulation and (3) the stimulated area (ROI vs. Control), by first normalizing the saccade latencies with the transformation log 1/RT where RT defined the reaction time or saccade latency. We excluded from theses analyses all the saccades in which the latencies were inferior to 100 ms. We performed a 3-way ANOVA on the normalized RTs using the three factors defined previously (Side, rTUS or the area) for each animal. For the timeline comparison of the mean latencies for no-rTUS and rTUS trials, we used a non-parametrical test with a correction for multiple comparisons (Benjamini and Hochberg, 1995).

## Results

### Global Behavioral Performance During Antisaccade Task

**FIGURE 2:**
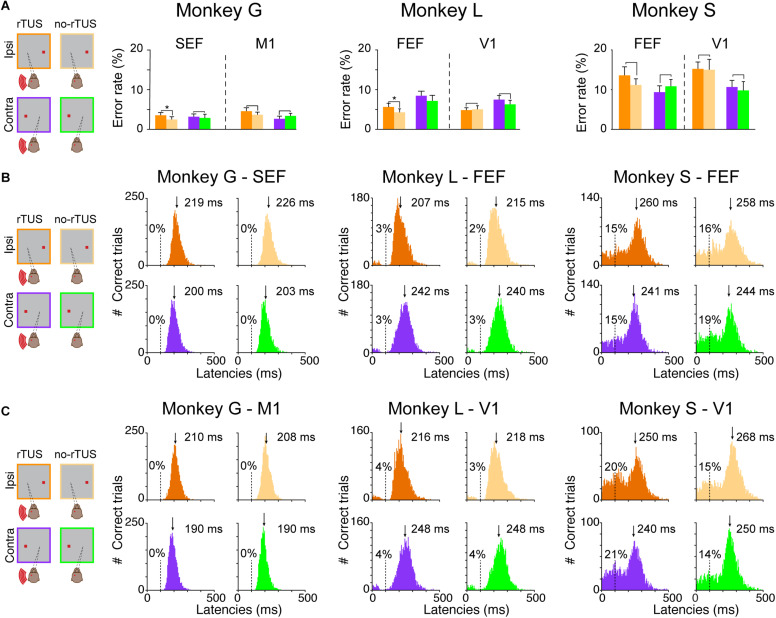
Behavioral performances during the antisaccade task. **(A)** Percentage of error rates for all three animals (monkey G; left quadrant, monkey L; middle quadrant and monkey S; right quadrant). Each bar represents the mean error rates for the post-rTUS blocks for the different conditions (violet/orange for contralateral vs. ipsilateral antisaccades, respectively, and dark/light and violet/green for rTUS vs. no-rTUS, respectively). Error bars indicate the standard error of the mean (SEM). We compared rTUS vs. no-rTUS mean error rates for each condition using a Wilcoxon rank sum test (*p < 0.05). Note that the performance for all conditions (ROI, Control, Contra, and Ipsi) were similar (*p* > 0.05) for all three animals except for monkeys G and L who made significantly more errors when rTUS was directed toward FEF/SEF regions for ipsilateral antisaccades. **(B,C)** Antisaccade latencies (ms) for all conditions [Contra/Ipsi, rTUS/no-rTUS for each animal (Monkeys G, L, and S), when the transducer was directed toward the oculomotor **(B)** or the control **(C)** areas]. The latencies were pooled over the pre-stimulation block (practice) and the next six post-rTUS blocks for each condition. The percentages indicate the percentage of anticipated saccades (<100 ms, dashed lines) over the total number of trials for each condition. For each histogram, the downside arrow indicates the median latency (ms) with the cutoff of anticipations.

All three monkeys performed the task across a total of 20 behavioral sessions for each targeted area (ROI or control) with an average error rate between 3 and 14% (Figure 2A). Monkey L and monkey G presented significantly higher error rates on the ipsilateral side of the FEF/SEF stimulation. Nevertheless, these increases in error were very low (monkey G from 2.5% to 3.5% for SEF ipsilateral side, and monkey L from 4.6 to 7.2% for FEF ipsilateral side), suggesting that the animals were performing the task uniformly during both rTUS and no-rTUS sessions. In addition, it suggests that rTUS did not disturb the animals’ general performance when either the frontal or the primary cortices were the targets of the neurostimulation. Monkey S, however, behaved differently and presented a higher error rate in all conditions (more than 9% for contra/ipsi side and control/tested areas). Our interpretation is that monkey S was using a different strategy with a higher guessing rate than the other animals for the antisaccade target location. In the context of antisaccades, a guessing strategy generates high error rates and often a bimodal latency distribution.

To confirm our theory, we analyzed the latency distributions of all monkeys (Figures 2B,C). The antisaccade latency for each individual trial was calculated as the time between the stimulus presentation and the onset of the eye saccade velocity (30°s^–1^). We compared saccade latencies between trials depending on different conditions: (1) whether it was a rTUS or no-rTUS session; (2) whether it was an ipsilateral or a contralateral antisaccade; and (3) whether the transducer was directed toward the oculomotor (FEF or SEF) or the control areas (V1 or M1). Figure 2B represents all the histograms for the different conditions (rTUS/no-rTUS: dark orange/violet vs. light orange/green; contra/ipsi: orange vs. violet/green; and ROI/Control: Figure 2B vs. Figure 2C) for all three animals.

Monkeys G and L both showed a unimodal distribution of the saccade latencies for ROI and control areas with very few anticipated saccades (i.e., saccade with latencies < 100 ms). Monkey G almost never anticipated the saccade (only 2 out of 26,821 trials) and monkey L had a relatively low number of anticipated saccades (<4%). In comparison, the distribution of saccade latencies for monkey S differed significantly presenting larger proportions of anticipated saccades (14–21%), suggesting that monkey S used a different strategy than the other two animals. The distribution of saccade latencies for monkey S confirmed that this animal was guessing and anticipating the appearance of the target.

We computed an ANOVA on normalized saccade latencies for each animal with the following main factors: the stimulation type (rTUS/no-rTUS), the side of the antisaccade (contra/ipsi) and the area (ROI/Control). Based on the ANOVA, we first reported the results of the main factors. We noticed that monkey G and monkey S showed an important lateralized bias for the contralateral side with saccades with significant shorter latencies (ANOVA, *p* < 0.001). For monkey L, a similar bias was present but only for the ipsilateral side, with saccades having significant shorter latencies (ANOVA, *p* < 0.001). Concerning the effect of rTUS stimulation, rTUS induced shorter latencies in all three animals (*p* < 0.001 for monkey G and monkey S, and *p* = 0.0064 for monkey L). Monkey S showed similar latencies for saccades when the transducer was directed toward FEF or the visual cortex (ANOVA, *p* = 0.192) although the saccades were made significantly earlier when rTUS was focused on the frontal areas (FEF/SEF) compared to the control sites (visual or motor cortex, ANOVA, *p* < 0.001). Concerning the interactions, for monkey G and monkey S, rTUS did shorten the latencies for contralateral and ipsilateral sides for both ROI and control areas (ANOVA, interaction Bonferroni, *p* < 0.05) although monkey L had no significant interaction for rTUS on saccade latencies.

### Sustained Effect of rTUS on Ipsilateral Anti-saccades

**FIGURE 3:**
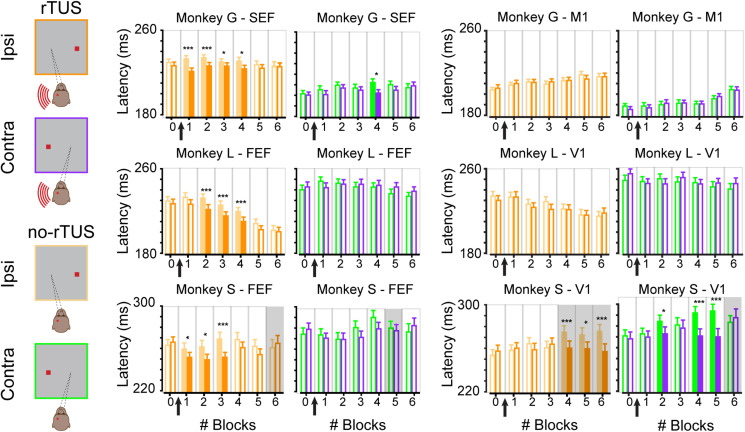
Time course of rTUS effect on mean latencies. Mean latencies per block when rTUS was directed toward the oculomotor areas (SEF/FEF; two left columns) or the control areas (M1/V1; two right columns) in three animals (monkey G; top row, monkey L; middle row and monkey S; bottom row). Each bar represents the mean latency averaged over all successful antisaccades in each block of trials for each side (contra/ipsi), error bars indicate SEM. Open bars indicate non-significant differences (Wilcoxon rank sum test, *p* > 0.05); filled bars indicate significant differences (* for *p* < 0.05 and *** for *p* < 0.001, corrected for multiple comparisons). Gray backgrounds indicate when error rates were superior to 15% although light backgrounds indicate when error rates were inferior to 15%. Black arrows schematize when rTUS was performed between blocks #0 and #1.

We showed that rTUS has a main effect of inducing shorter saccades. We now wanted to investigate how long the effect of rTUS lasted. Each animal performed a complete block of trials before rTUS stimulation (block #0). We then collected the saccade latencies during six additional blocks of trials (block #1 to block #6). The timeline of the mean latencies for all three monkeys for ipsilateral and contralateral antisaccades are displayed in Figure 3. We compared for each block of trials the no-rTUS and r-TUS saccades latencies with a non-parametric Wilcoxon test using multiple corrections (Benjamini and Hochberg, 1995). For monkey G, a significant effect of rTUS was observed in the first four blocks of trials for ipsilateral antisaccades only when the transducer was directed toward frontal regions (Figure 3, monkey G – SEF). For monkey L, rTUS showed no effect in the first block of trials (block #1) but was significant in the next three blocks (block #2 to block #4) with shorter latencies saccades on the ipsilateral side. On average, four blocks of trials correspond to a duration of between 21 and 30 min (mean ± SEM; 21 min ± 1 for monkey G and 30 min ± 0.4 for monkey L). After this time period, the saccade latencies were statistically indistinguishable from the latencies recorded in the sham condition (no-rTUS). For contralateral trials, only a few sporadic blocks of trials showed significantly shorter latencies (block #4 for monkey G and none for monkey L) for the rTUS condition. No significant behavioral change was seen when the transducer was directed toward the control regions (Figure 3, control areas; M1 and V1). For both monkey G and monkey L, as seen in Figure 2, we did not notice any difference in any of the six post-rTUS blocks for both contralateral and ipsilateral trials (Wilcoxon rank sum test, *p* > 0.05). These results confirm that rTUS during an antisaccade task only affect the behavioral performance of the animals when targeted in their frontal cortex and not their visual/motor cortex.

We then wanted to better understand the behavioral performance of monkey S as we noticed that, contrary to the two others monkeys, monkey S performed significantly more anticipated saccades, suggesting the use of a different strategy. We focused our analysis on saccade latencies that were superior to 175 ms. We choose this threshold based on the histograms of the saccade latencies for monkey S (Figures 2B,C) as they were more similar to the ones for the other animals (even though approximately a third of the saccades were excluded from this analysis, 30% for the FEF neuronavigated rTUS sessions, and 38–39% for V1 neuronavigated rTUS sessions). We next wanted to check whether the effect of neuronavigated rTUS on early blocks after rTUS was present in these antisaccades. Similarly to the two other animals, the analyses of the time courses per block (Figure 3 bottom panel) showed a significant decrease of mean latencies for ipsilateral antisaccades when the transducer was directed toward the FEF locations for the first blocks of trial post-rTUS (block #1 to block #3; 18 min ± 0.5). No variations on ipsilateral saccades on early blocks were noticed with the visual cortex stimulations. However, monkey S showed significant differences for late blocks (block #4 to block #6) when the transducer was directed toward V1 triggering slower saccades for the no-rTUS sessions. Indeed, the mean latencies for these three last blocks of trials increased significantly compared to block #0. Moreover, monkey S showed poor performance during these blocks of trials (error rate > 15%). We also noticed slower latencies for late blocks for contralateral saccades for V1 stimulation sessions (blocks #2, #4, and #5).

## Discussion

This study demonstrates the feasibility of using focused ultrasound to modulate visual behavior for a sustained period of several minutes in the awake non-human primate brain. Here we show that continuous low frequency (320 kHz) and low-pressure amplitude (estimated at 0.4 MPa *in situ* corresponding mechanical index of 0.7) pulses of ultrasound lasting 20 s can be used in order to achieve these long-lasting effects. We have demonstrated that rTUS set with such parameters, applied to oculomotor regions of the frontal cortex, produces a subtle significant shortening of ipsilateral saccade latencies and a subtle increase of error rates, as compared to non-stimulated trials. Together, these results show that neuronavigated rTUS applied to oculomotor regions can have a sustained effect on saccades when they occur on the ipsilateral side of stimulation.

### Single vs. Repetitive Stimulation

Compared to the modulation of response times reported with repetitive Transcranial Magnetic Stimulation (rTMS), our results appear to be less affected by baseline variability of response times as no normalization was required (Thut and Pascual-Leone, 2009). In addition, no discomfort (muscle twitching) was observed during ultrasonic neurostimulation, in contrast to that observed during rTMS sessions. In previous studies, while stimulating FEF, saccadic latencies after single pulses of TUS or TMS have been reported to be significantly delayed (Grosbras and Paus, 2002; Deffieux et al., 2013). As described in this study, the use of repetitive stimulation facilitates antisaccade triggering. These results are in accordance with findings observed following rTMS, demonstrating facilitation effects (Crevits et al., 2005) while compared to single pulse TMS (Olk et al., 2005). We propose that repetitive stimulation may significantly interfere, compared to single pulse, with the processing of inhibition, which is particularly active in antisaccade tasks (Everling and Munoz, 2000). Although the exact mechanisms are still unknown, ultrasonic neurostimulation is believed to be mechanical rather than thermal, as illustrated by experimental evidence on rodents, where lower frequencies yield smaller motor thresholds (Ye et al., 2016). Also, we speculate that our results following rTUS over primary visual cortex may interfere with the processing of visual activity in the superior colliculi and therefore may provoke an increased rate of anticipated saccades (here, observed with monkey S who performed a high rate of saccades with short latencies).

Altogether, our results support the use of this approach to study brain function and non-invasive neurostimulation for exploratory and therapeutic purposes with unprecedented spatial resolution. The spatial resolution is proportional to the ultrasonic wavelength (1.5 mm at 1 MHz) and decreases with the ultrasonic frequency. Increasing the frequency thus improves the precision of the stimulation but, unfortunately, it also induces a higher thermal rise due to increased tissue absorption (Constans et al., 2018). Furthermore, aberrations induced by the skull increase with frequency (Maimbourg et al., 2018b; Leung et al., 2019). Therefore, targeting brain regions non-invasively is easier with low frequency ultrasound (Yin and Hynynen, 2005). For frequencies higher than 300 kHz, CT-based (Marquet et al., 2009; Pulkkinen et al., 2014) or MR-based (Wintermark et al., 2014; Miller et al., 2015) aberration correction needs to be performed. This can be achieved with multielement arrays of transducers (Jeanmonod et al., 2012; Chauvet et al., 2015) or with single element transducers combined with an acoustic lens (Maimbourg et al., 2018a, 2019). With these latest developments, rTUS appears to be very promising and offers a novel and competitive neurostimulation technique. When compared to rTMS, rTUS offers a higher spatial resolution (Pinton et al., 2012), a larger targeting envelope (Krishna et al., 2018) and the absence of noise or mechanical vibration during stimulation (Mohammadjavadi et al., 2019). This technique shows promise in the non-invasive exploration of cognition with non-human primate models. Used with ultrasound contrast agents (Magnin et al., 2015; Pouliopoulos et al., 2019), neuronavigated rTUS can be used to deliver allosteric modulators locally as well as to transiently modulate brain activity (McDannold et al., 2015; Constans et al., 2019).

### Application on Humans

Although the exact mechanisms of focused ultrasound neurostimulation are still unknown, the ability to modulate precisely defined deep brain structures non-invasively opens exciting possibilities in the treatment of psychiatric and neurological disorders in humans, such as treatment-resistant depression, anxiety disorders, Parkinson’s disease, essential tremor, and disorders of consciousness. The promise of TUS also lies in its unique ability to explore safely brain circuitry and mechanisms underlying complex cognitive functions, as our team has previously demonstrated in non-human primates (Fouragnan et al., 2019; Khalighinejad et al., 2020).

In current rTMS clinical trials, neuronavigation can be seen as a necessary add-on to take advantage of inter-subject anatomical variability and allow more precise targeting (Fitzgerald et al., 2009; Dollfus et al., 2017). Even though several cortical-rTUS human studies have used non-navigated procedures (Mueller et al., 2014; Sanguinetti et al., 2020), neuronavigation should be seen not only as a valuable tool in deep-rTUS, but also a requirement. Indeed, given its millimeter accuracy and its capability of hitting deep targets (without stimulating unintended brain structures dorsal to stimulation), neuronavigated rTUS may be the ideal non-invasive deep brain stimulation tool. These two innovations have already been coupled in several proof-of-concept human studies in healthy volunteers (Legon et al., 2018a, b) and patients (Brinker et al., 2020). Interesting is the fact that clinicians are now using fMRI and neuronavigated rTMS to modulate different target areas in the brain of patients depending on the neural circuits of these individuals (Siddiqi et al., 2020). The use of fMRI, along with navigated rTUS in humans, may enhance even more our ability to provide personalized and supportive-based neuromodulation therapy. Our team took advantage of this technique and used fMRI data along with navigated rTUS in non-human primates (Khalighinejad et al., 2020). Like any new technique, the safety of the ultrasound parameters needs to be further investigated before translating these novel stimulation protocols to the clinic. In particular, ultrasound induces a higher thermal rise at higher frequencies due to increased tissue absorption (Duck, 1990). Numerical models estimating the thermal rise in tissues has been developed (Constans et al., 2018) and experimentally validated (Ozenne et al., 2019) to help design novel TUS systems. The safety of ultrasonic neurostimulation has additionally been assessed by histology in rodents (Yoo et al., 2011; Yang et al., 2012; Mehić et al., 2014; Li et al., 2016), pigs (Dallapiazza et al., 2018), sheep (Gaur et al., 2020) and monkeys (Blackmore et al., 2019; Verhagen et al., 2019; Gaur et al., 2020).

## Conclusion

This study demonstrates that long lasting (20 s) repetitive transcranial ultrasound stimulation modulates visual behavior of awake non-human primates for a sustained period of several minutes. rTUS was specifically targeted to the Frontal Eye Field and the Supplementary Eye field with a frameless neuronavigation system. Neuronavigated rTUS holds promise in exploring brain circuitry by offering novel treatment modalities based on sustained neuromodulation.

## Data Availability Statement

The datasets presented in this study can be found in online repositories. The names of the repository/repositories and accession number(s) can be found below: The raw data supporting the conclusions of this article will be made available by the authors, without undue reservation.

## Ethics Statement

The animal study was reviewed and approved by the recommendations of the EEC (86/609) and the French National Committee (87/848). Our experiments were authorized by the Animal Health and Veterinary Medication Division of the Department of Public Veterinary Health, Nutrition and Food Safety of the French Ministry of Health (last renewal N°: DTPP 2010-424).

## Author Contributions

J-FA and PP designed the study. HA, JC, and CC performed the animal experiments. J-FA, CC, and DA performed the acoustic calibrations. SF, PP, and HA set up the neuronavigator. FA, HA, and PP analyzed the data. FA, PP, SF, DA, and J-FA wrote the manuscript with input from all authors. All authors contributed to the article and approved the submitted version.

## Conflict of Interest

SF was a full time employee and partner of Rogue Research Inc. The remaining authors declare that the research was conducted in the absence of any commercial or financial relationships that could be construed as a potential conflict of interest.
